# The influence between frailty, sarcopenia and physical status on mortality in patients undergoing emergency laparotomy

**DOI:** 10.1186/s13017-025-00588-5

**Published:** 2025-04-30

**Authors:** May Myat Thu, Hwei Jene Ng, Susan Moug

**Affiliations:** 1 School of Medicine, University of Glasgow, University Place, Glasgow, G12 8QQ UK; 2 Department of General Surgery, Royal Alexandra Hospital, Corsebar Road, Paisley, PA2 9PN UK

**Keywords:** Sarcopenia, Frailty, Poor physical status, Emergency laparotomy

## Abstract

**Background:**

Frailty and sarcopenia have been independently shown to predict mortality in emergency laparotomy (EmLap), and both can be indicative of poor physical status. We aim to assess the prevalence of frailty, sarcopenia, and physical status in EmLap and explore the relationship between these factors and 30-day, 90-day and 1-year mortality.

**Methods:**

Retrospective analysis was performed on prospectively maintained Emergency Laparotomy and Laparoscopic Scottish Audit (ELLSA) database (2017–2019) which included patients ≥ 18 years who underwent EmLap. Clinical frailty scale (CFS) was used to classify frailty (score ≥ 4 as frail). Sarcopenia was assessed using total psoas index (TPI). Poor physical status (PPS) was defined by American Society of Anaesthesiologists physical status classification (ASA) ≥ 4. Binary logistic regression and fisher’s exact tests were used for statistical analysis.

**Results:**

215 patients were included in the study, with 57.2% female and median age of 64 years. Frailty was present in 17.2%, sarcopenia in 25.1% and 14.4% had PPS; 3.3% had all three factors. Frail patients had significantly higher risk for 30-day (*p* = 0.003), 90-day (*p* = 0.006) and 1-year mortality (*p* = 0.032). Patients with poor physical status also showed significantly higher mortality at 30-day (*p* < 0.001), 90-day (*p* < 0.001) and 1-year (*p* = 0.001). Sarcopenic patients did not show significant differences in mortality risks up to 1 year. Patients with all three factors had significantly higher 30-day (*p* = 0.003), 90-day (*p* = 0.046) and 1-year mortality (*p* = 0.108) compared to patients who had none of the factors.

**Conclusions:**

Frailty, sarcopenia, and PPS are prevalent in EmLap. Frailty and PPS were independently associated with short and long-term mortality, but not sarcopenia. While overlap exists between three factors, more research is required to understand the complex interplay.

**Supplementary Information:**

The online version contains supplementary material available at 10.1186/s13017-025-00588-5.

## Introduction

The UK Emergency Laparotomy and Frailty (ELF) study in 2021 reported that 20% of older adults undergoing emergency laparotomy (EmLap) were frail. The ELF study demonstrated that, independent of age, higher frailty scores were associated with increased risks of post-operative 30-day and 90-day mortality and morbidity, and increased dependency after discharge [1, 2]. Consequently, National Emergency Laparotomy Audit (NELA), the world’s largest prospective database for EmLap which primarily collects data from England and Wales, incorporated frailty scoring into their required database. Smart et al. highlighted that frailty affects 16% of adults from 40 to 64.9 years admitted for surgical emergencies, emphasizing the broader impact beyond the older age group [3]. Irrespective of age, the understanding of mechanism of frailty remains poorly understood, highlighting a clear need for further research in this domain.

Frailty is a multidimensional syndrome characterised by increased vulnerability due to accumulation of physiological deficits in multiple systems as people age [4]. Part of this syndrome includes sarcopenia, characterised by progressive and generalised loss of skeletal muscle mass and physical function. A meta-analysis by Humphry et al. showed the median rate of incidence of sarcopenia was around 25.1% in patients undergoing EmLap [5]. Studies have found sarcopenia to be predictive of short and long-term outcomes in elective intra-abdominal surgeries [6–9], and findings were mirrored in the EmLap settings [10]. 

In predicting operative risks and perioperative outcomes, the American Society of Anaesthesiologist classification (ASA score) is also commonly used. Here, the patient’s physical status is categorised into 6 subgroups (ASA 1–6) [11] and numerous studies have shown strong correlation between high physical classification status and early post-operative death [12–14]. It is also found to be a good discriminator of long-term outcome following EmLap [15]. 

While existing studies have determined the prevalence of frailty, sarcopenia, or physical status, and their respective impact on post-operative outcomes in emergency settings, there are few studies investigating all three conditions at once. This study primarily aims to define the prevalence of frailty, sarcopenia, and physical status in all adults undergoing emergency laparotomy. The secondary aim was to explore the relationship between frailty, sarcopenia, and physical status in regard to 30-day, 90-day and 1-year mortality.

## Methodology

Data was extracted from the prospectively maintained Emergency Laparotomy and Laparoscopic Scottish Audit (ELLSA) databases from two acute surgical hospitals, between November 2017 and January 2020. ELLSA is one of several Scottish Government initiatives in the Modernising Patient Pathway Programme aimed at supporting local teams in coordinating and understanding emergency laparotomy patient care across NHS Scotland [16]. Approval was given by NHS National Services Caldicott Guardian Greater Glasgow and Clyde. Patient identifiable information was anonymised before data transmission, therefore individual patient consent was not necessary.

The inclusion and exclusion criteria for ELLSA is based on the NELA criteria [17] (https://www.nela.org.uk/criteria).

All adults aged 18 and over were included with age, sex, and type of operation recorded. In addition to the NELA exclusion criteria, patients were also excluded in the preoperative CT scan was more than 10 days prior to EmLap to ensure an up-to-date measurement of sarcopenia, or if no frailty or ASA score were recorded. Patient’s electronic records were followed up for 30-day, 90-day and 1-year mortality after the date of surgery.

Psoas major muscle has been demonstrated to be an accurate marker of overall skeletal mass and predict outcomes in wide range of surgical specialties. Although there is a broad agreement over the predictive value of psoas major measurement, there is a lack of consensus and variance with respect to optimal cut-off values and sex-specific values defining sarcopenia and optimal method of measurement. This study follows several pre-existing studies studying sarcopenia in EmLap in using Total Psoas Muscle Index (TPI) [18–21]. 

**Fig. 1:**
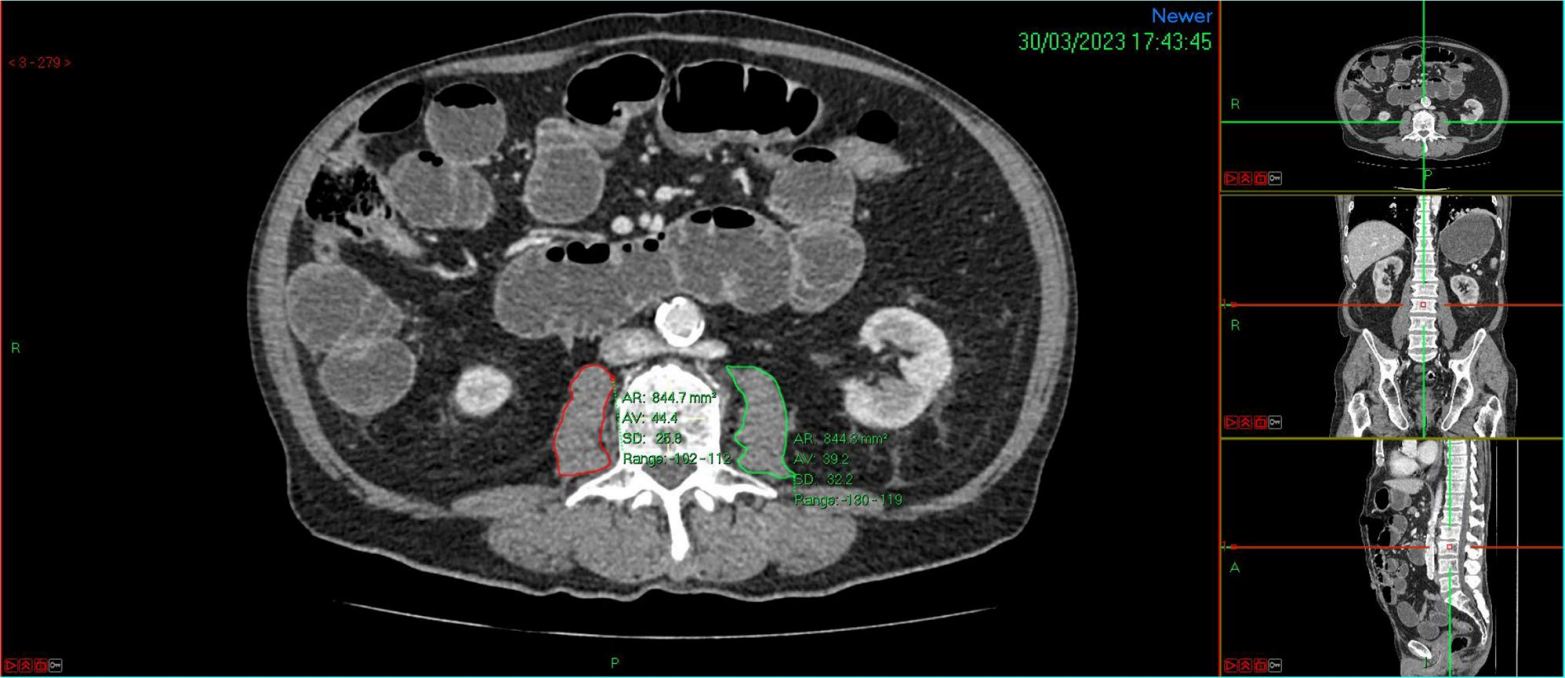
CT Abdomen and Pelvis, at the level of L3 vertebra, demonstrating method of measuring total psoas area (TPA). Outlines of left (green) and right (red) psoas major muscle were manually traced on PACS

Patients were sex-stratified and split into quartiles based on their TPI. (Fig. 1) In line with previous studies [18–21], the lowest quartile for each sex was identified as ‘sarcopenic’ while the remaining three quartiles were labelled as ‘not sarcopenic’ and used for comparison. The thresholds calculated for sarcopenia in this study population were 475.48 mm^2^/m^2^ for males and 345.14 mm^2^/m^2^ for females. The inter- and intra-class correlation coefficients (ICCC) were calculated to assess the reliability of sarcopenia measurement technique. 20 scans were randomly selected and TPA was measured by blinded and trained investigators. The ICCC values for inter- and intra-class reliability were 0.934 and 0.928, respectively. Values close to 1 indicate good agreement. *(*Additional file Fig. 1)

Rockwood Clinical Frailty Scale (CFS) was used as a measure for frailty, classified as 1–3 = not frail, 4 = pre-frail and 5–7 = frail.[22] Due to predicted low numbers of patients with a frailty score 5–7, pre-frail patients were grouped together with frail patients into a single frail category for analysis. In September 2020, an updated version of the CFS was introduced, extending the levels from 1 to 9 [23]. The data in this study reflects the earlier version and reflects the frailty scoring used at that time.

American Anaesthesiologist physical status classification (ASA) was used to determine physical status, with poor physical status (PPS) defined as ASA ≥ 4.

### Statistical analysis

Data was reported as median and interquartile range (IQR), for continuous variables [IBM Corp. Released 2021. IBM SPSS Statistics for Windows, Version 28.0. Armonk, NY: IBM Corp]. The remaining data was reported using numerical figures and percentages. Binary logistic regression was used to compare 30-day, 90-day and 1-year mortality between frail and non-frail cohorts, sarcopenic and non-sarcopenic cohorts, and poor and normal physical status. Fisher’s exact test was used to compare 30-day, 90-day and 1-year mortality for patients with all three factors and patients with none of the factors.

## Results

### Patient demographics, pathological and physiological characteristics

**Fig. 2:**
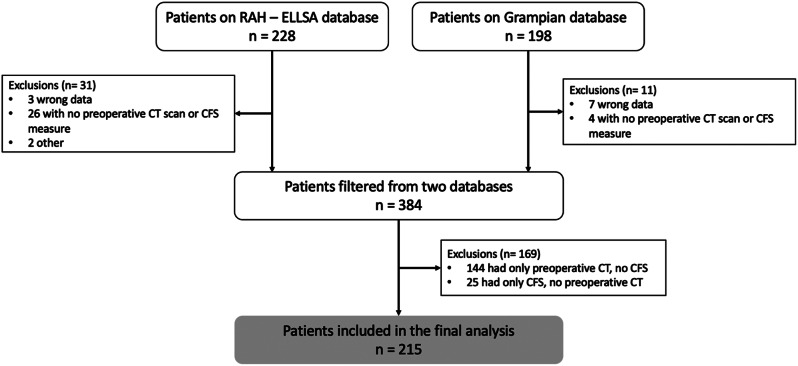
Patient flowchart for the study, and reasons for exclusion. The Strengthening the Reporting of Observational studies in Epidemiology (STROBE) guidelines were followed

Of 426 patients undergoing EmLap, 211 patients were removed after applying exclusion criteria of missing key data (Fig. 2).

This resulted in 215 eligible patients, with median age of 64 years (IQR 50–73, range 18–91) and 57.2% being female. There were 107 (49.8%) patients aged 65 years and above. The most common EmLap procedure was right colectomy (19.5%) and small bowel resection (19.1%), followed by Hartmann’s procedure (15.8%). (Additional file Table 1)

The overall 30-day mortality rate was 3.7% (8/215), 90-day mortality was 7.4% (16/215) and 1-year mortality was 11.2% (24/215).

**Fig. 3:**
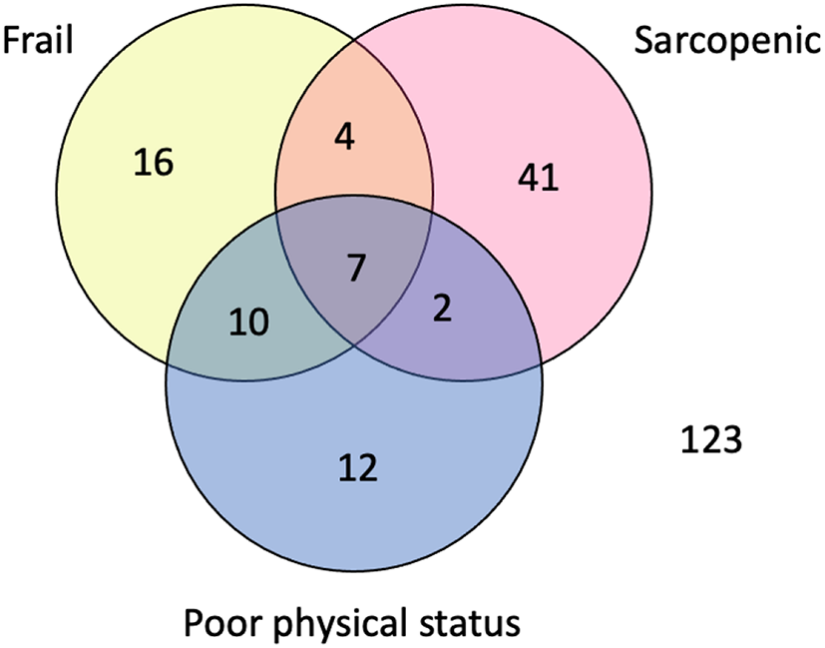
Venn-Diagram showing distribution of patients with frailty, sarcopenia, and poor physical status

In this cohort, frailty was present in 17.2% of the patients, 25.1% were sarcopenic and 14.4% had poor physical status (Table 1). 16 (7.4%) were only frail, 41 (19.1%) were only sarcopenic, 12 (5.6%) only had poor physical status, and 7 (3.3%) had all three factors, and 122 (56.7%) had none of the factors (Fig. 3). The overall mortality for each subgroup can be found in Table 2.

**Table 1:** Demographics, characteristics and 30-day, 90-day and 1-year mortality of patients who underwent emergency laparotomy (total cohort *n* = 215)

Characteristic	*n* (%)
*Sex*	
Male	92 (42.8%)
Female	123 (57.2%)
*Age*	
Median age, years (IQR)	64 (50–73)
Age $\:\ge\:$ 65years	107 (49.8%)
*ASA*	
1– Normal healthy	31 (14.5%)
2– Mild systemic disease	76 (35.5%)
3– Severe systemic disease	77 (35.8%)
4– Severe systemic disease that is constant threat to life 5– Moribund, not expected to survive without operation	30 (14.0%) 1 (0.5%)
Normal physical status– ASA 1 to 3	184 (85.6%)
Poor physical status– ASA 4 to 5	31 (14.4%)
*Clinical Frailty Scale (CFS)*	
Median CFS (IQR)	2 (2–3)
1– Very fit	53 (24.7%)
2– Fit	70 (32.6%)
3– Managing well	55 (25.6%)
4– Very mild frailty	28 (13.0%)
5– Mild frailty	5 (2.3%)
6– Moderately frail	2 (0.9%)
7– Severely frail	2 (0.9%)
Not Frail– CFS 1 to 3	178 (82.8%)
Frail– CFS 4 to 7	37 (17.2%)
*Total Psoas Index (TPI)*	
Median TPI, mm^2^/m^2^– Male (IQR)	606.75 (475.48–750.30)
Median TPI, mm^2^/m^2^– Female (IQR) Non-sarcopenic Sarcopenic	437.12 (345.14–533.58) 161 (74.9%) 54 (25.1%)
*Overall mortality*	
Overall 30-day mortality Overall 90-day mortality Overall 1-year mortality	8 (3.7%) 16 (7.4%) 24 (11.2%)

ASA = American Society of Anaesthesiologists Physical Status Classification

**Table 2:** Overall 30-day, 90-day and 1-year mortality rates for each subgroup

	30-day mortality *n* (%)	90-day mortality *n* (%)	1-year mortality *n* (%)
Frail (*n* = 37)	5 (13.5%)	7 (18.9%)	8 (21.6%)
Non-frail (*n* = 178)	3 (1.7%)	9 (5.1%)	16 (9.0%)
Sarcopenic (*n* = 54)	2 (3.7%)	3 (5.6%)	6 (11.1%)
Non-sarcopenic (*n* = 161)	6 (3.7%)	13 (8.1%)	18 (11.2%)
Poor physical status (*n* = 31)	7 (22.5%)	8 (25.8%)	9 (29.0%)
Normal physical status (*n* = 184)	1 (0.5%)	8 (4.3%)	15 (8.2%)
All three factors (*n* = 7)	2 (28.6%)	2 (28.6%)	2 (28.6%)
None of the factors (*n* = 123)	0 (0%)	5 (4.1%)	9 (7.3%)

### 30-day, 90-day and 1-year mortality in frail, sarcopenic, and poor physical status respectively

Frail patients had higher mortality than non-frail patients at 30-days with odds ratio (OR) 9.12 (95% CI: 2.08–40.05, *p* = 0.003). Results remained significant after adjusting for age and for both age and sex. Similar results were obtained for 90-day mortality (OR 4.38, 95% CI: 1.52–12.66, *p* = 0.006) and 1-year mortality (OR 2.79, 95% CI: 1.10–7.12, *p* = 0.032). However, results were not significant after adjusting for age, and for both age and sex (Table 3).

Sarcopenic patients did not vary in mortality rates compared to non-sarcopenic patients at 30-days with OR 1.19 (95% CI: 0.23–6.33, *p* = 0.836) and remained non-significant after adjusting for age, then with age and sex. Similar results were obtained for 90-day and 1-year mortality (Table 3).

Patients with poor physical status had higher mortality than patients with normal physical status at 30-days with OR 53.38 (95% CI: 6.29–452.78, *p* < 0.001), 90-days with OR 7.65 (95% CI: 2.62–22.35, *p* < 0.001) and 1-year with OR 4.61 (95% CI:1.80–11.78, *p* = 0.001). Both short-term and long-term mortality results remained significant after adjusting for age and age and sex (Table 3).

**Table 3:** Frailty, Sarcopenia and Poor Physical Status in association with 30-day, 90-day and 1-year mortality using Binary Logistic Regression

*30-day mortality*			
Patient group	Frail vs. Non-Frail	Sarcopenic vs. Non-sarcopenic	Poor Physical Status vs. Normal Physical Status
Crude odds ratio (95% CI)	9.12 (2.08–40.05)	1.19 (0.23–6.33)	53.38 (6.29–452.78)
p-value	**0.003**	0.836	**< 0.001**
Adjusted odds ratio– age (95% CI)	5.96 (1.15–30.9)	1.03 (0.19–5.57)	43.38 (5.03–372.17)
p-value	**0.034**	0.971	**< 0.001**
Adjusted odds ratio– age & sex (95% CI)	6.18 (1.27–35.51)	1.02 (0.19–5.53)	48.25 (5.41–430.25)
p-value	**0.025**	0.986	**< 0.001**
*90-day mortality*			
Crude odds ratio (95% CI)	4.38 (1.52–12.66)	0.73 (0.20–2.67)	7.65 (2.62–22.35)
p-value	**0.006**	0.630	**< 0.001**
Adjusted odds ratio– age (95% CI)	2.68 (0.84–8.55)	0.62 (0.17–2.34)	6.18 (2.07–18.45)
p-value	0.096	0.484	**0.001**
Adjusted odds ratio– age & sex (95% CI)	2.84 (0.88–9.20)	0.62 (0.16–2.32)	6.27 (2.10–18.73)
p-value	0.082	0.476	**0.001**
*1-year mortality*			
Crude odds ratio (95% CI)	2.79 (1.10–7.12)	1.05 (0.39–2.82)	4.61 (1.804–11.78)
p-value	**0.032**	0.921	**0.001**
Adjusted odds ratio– age (95% CI)	1.78 (0.64–4.92)	0.93 (0.34–2.53)	3.80 (1.46–9.92)
p-value	0.268	0.885	**0.006**
Adjusted odds ratio– age & sex (95% CI)	1.83 (0.66–5.10)	0.92 (0.34–2.52)	3.81 (1.46–9.92)
p-value	0.249	0.878	**0.006**

### Relationship between frailty, Sarcopenia and poor physical status

Patients with all three factors compared to patients who had none of the factors had higher mortality at 30-days (*p* = 0.003), 90-days (*p* = 0.049) and 1-year (*p* = 0.108). (Table 4)

**Table 4:** Comparing mortality in patients with all three factors vs. patients with none of the factors using Fisher’s exact test

	All three factors (*n* = 7)	None of the factors (*n* = 123)
30-day mortality (n)	2	0
Percentage proportion	28.6%	0%
p value	**0.003**	
90-day mortality	2	5
Percentage proportion	28.6%	4.1%
p value	**0.046**	
1-year mortality	2	9
Percentage proportion	28.6%	7.3%
p value	0.108	

Analysis was performed on 4 different subgroups– “Frail and Sarcopenic”, “Frail and Poor Physical Status”, “Sarcopenic and Poor physical status” and “All three factors”. Higher 30-day mortality was found for patients in all 4 subgroups (*p* = 0.024, *p* < 0.001, *p* = 0.013, *p* = 0.005) respectively. There was a higher risk of 90-day mortality in patients with both frailty and poor physical status (OR 7.08, 95% CI: 2.12–23.70, *p* = 0.001), which remained significant when adjusted for and adjusted for age and sex. No other subgroups had statistically significant difference for 90-day mortality. Higher 1-year mortality was found for patients with both frailty and poor physical status (OR 3.93, 95% CI: 1.25–12.34, *p* = 0.019), and for patients with both sarcopenia and poor physical status (OR 4.41, 95% CI: 1.03–18.92, *p* = 0.046), but results were not significant after adjusting for age, and for age and sex (Table 5).

**Table 5:** Comparing 30-day, 90-day and 1-year mortality in patients with overlapping factors

	30-day mortality	90-day mortality	1-year mortality
“Frail & Sarcopenic” vs. “Not Frail & Sarcopenic”			
Crude odds ratio (95% CI)	7.33 (1.30–41.54)	3.02 (0.59–15.32)	1.84 (0.37–9.06)
p value	**0.024**	0.183	0.454
Adjusted for age (95% CI)	4.98 (0.84–29.63)	2.05 (0.39–10.86)	1.30 (0.26–6.64)
p value	0.078	0.399	0.749
Adjusted for age & sex (95% CI)	6.08 (0.95–39.04)	2.21 (0.41–11.97)	1.35 (0.26–6.96)
p value	0.057	0.358	0.720
“Frail & Poor Physical Status” vs. “Not Frail, & Normal Physical Status”			
Crude odds ratio (95% CI)	14.92 (3.35–66.57)	7.08 (2.12–23.70)	3.93 (1.25–12.34)
p value	**< 0.001**	**0.001**	**0.019**
Adjusted for age (95% CI)	10.09 (2.14–47.64)	4.78 (1.37–16.67)	2.74 (0.84–8.94)
p value	**0.003**	**0.014**	0.094
Adjusted for age & sex (95% CI)	12.99 (2.52–66.88)	5.27 (1.47–18.82)	2.87 (0.87–9.43)
p value	**0.002**	**0.014**	0.084
“Sarcopenic & Poor Physical Status” vs. “Not Sarcopenic, & Normal Physical Status”			
Crude odds ratio (95% CI)	9.52 (1.62–55.85)	3.92 (0.74–20.66)	4.41 (1.03–18.92)
p value	**0.013**	0.107	**0.046**
Adjusted for age (95% CI)	7.17 (1.18–43.58)	2.93 (0.54–15.95)	3.43 (0.78–15.09)
p value	**0.032**	0.214	0.094
Adjusted for age & sex (95% CI)	7.98 (1.28–49.67)	3.04 (0.56–15.58)	3.48 (0.79–15.32)
p value	**0.026**	0.199	0.099
“All three factors” vs. “Not Frail, Sarcopenic, & has Normal Physical Status”			
Crude odds ratio (95% CI)	13.47 (2.16–83.94)	5.54 (0.98–81.78)	3.38 (0.62–18.48)
p value	**0.005**	0.052	0.160
Adjusted for age (95% CI)	9.76 (1.50–63.38)	4.00 (0.69–23.345)	2.51 (0.45–14.09)
p value	**0.017**	0.123	0.296
Adjusted for age & sex (95% CI)	14.24 (1.92–105.54)	4.57 (0.76–27.52)	2.67 (0.47–15.23)
p value	**0.009**	0.097	0.270

### Prevalence of frailty, Sarcopenia and poor physical status between younger adults (age < 65) and older adults (age ≥ 65) subgroups

The study included 108 (50.2%) younger adults (patients aged under 65). Of this cohort, 5 (4.6%) were frail, 24 (22.2%) were sarcopenic and 9 (8.3%) had poor physical status. The overall mortality in younger adult population for 30-day, 90-day and 1-year were 1.9%, 3.7% and 3.7% respectively (Table 6).

Of 107 (49.8%) older adults (patients aged over 65), 30 (28.7%) were found frail, 32 (29.9%) were found sarcopenic, and 22 (20.6%) patients had poor physical status. The overall mortality in this patient population for 30-day, 90-day and 1-year were 5.6%, 7.5% and 9.3% respectively (Table 6).

**Table 6:** Comparing prevalence of frailty, Sarcopenia, poor physical status, and the difference in 30-day, 90-day, 1-year mortality rates between older adult (≥ 65 year old) and younger adult (< 65 year old) patients

Characteristic	Older adult (≥ 65 year old) *n* = 107	Younger adult (< 65 year old) *n* = 108
*Frail*		
CFS (4–7)	32 (29.9%)	5 (4.6%)
*Sarcopenic*	30 (28.3%)	24 (22.2%)
*Poor Physical Status*		
ASA ≥ 4	22 (20.6%)	9 (8.3%)
*Overall Mortality*		
30-day 90-day 1-year	6 (5.6%) 8 (7.5%) 10 (9.3%)	2 (1.9%) 4 (3.7%) 4 (3.7%)

## Discussion

In patients undergoing emergency laparotomy, there were high levels of frailty and sarcopenia, with frailty impacting 17.2% of patients, sarcopenia affecting 25.1% and 14.1% having poor physical status (defined by an ASA score of 4 or higher). The prevalence of frailty and sarcopenia found in this study are consistent with the findings of other studies which have investigated the prevalence of these factors separately in the EmLap cohort [1, 24, 25]. Poor physical status which found to predict both short and long-term mortality, irrespective of age and sex, was also found to be consistent with findings of other studies with reported association of co-morbidities and higher mortality rates in emergency surgery [26, 27]. Frail patients and patients with poor physical status demonstrated considerably elevated risks for 30-day, 90-day and 1-year mortality compared to non-frail patients and patients with normal physical status. This finding reflects existing studies and literature reviews which supports frailty as a strong predictive factor for mortality and morbidity in emergency settings [28–31]. This highlights the importance of frailty assessment in adults of all ages in the emergency settings as it is vital in aiding shared decision making preoperatively, prompting early engagement with allied healthcare professionals post-operatively and activating frailty-specific clinical pathways to provide the best, most appropriate, and supportive patient-centred care.

Frailty assessment was found lacking in the EmLap setting, which was demonstrated in this study where 174/426 (40.8%) of the patients were excluded due to absent CFS. This finding mirrored the Fifth NELA report where only 19% of the patients over 65 had formal frailty status assessed [32]. According to a recent 2022 survey by Knight T et al., two fifths of participating UK hospitals did not have a routine frailty screening policy, and when one did exist, rates of assessment of frailty were inconsistent and most at-risk patients were not screened [33]. In order to encourage and improve assessment of frailty in the EmLap settings, Rockwood Clinical Frailty Scale (CFS) has been integrated in the NELA as it is a validated and pragmatic method in assessing frailty in the emergency setting. As shown in the most recent eight NELA report, frailty assessment in adults over 65 years has improved drastically to 86.5% [34]. This reflects the feasibility of incorporating CFS routinely when assessing patients in emergency settings.

Majority of the patients included in this study had a CFS of 1–3 (Very Fit to Fit), while few patients were categorised into CFS 5–7 (Frail to Severely Frail). When considering risks and benefits of undergoing EmLap, the severely frail patients may choose non-surgical management or palliative care, as indicated by our findings. A newly proposed study hypothesises that frailty levels may change post-EmLap in both frail and non-frail patients, regardless of age, suggesting that frail patients may experience worse survival and quality of life compared to their non-frail counterparts [35]. In such cohort of patients, targeted strategies post-operatively, and early involvement of palliative team should be considered to facilitate symptom control and discharge planning as per patient and family’s wishes [36]. 

Sarcopenia has been shown to be predictive of mortality and morbidity in patients undergoing elective intra-abdominal operations [6–9]. Sarcopenia assessment can be done by measurement of psoas muscle attenuation on routine pre-operative CT scan. Compared to frailty scoring which has a low completion rate, 55/426 (12.9%) of the patients in this study did not have pre-operative CT scan. This is consistent with the Eighth NELA report which states 91.8% of patients requiring EmLap underwent pre-operative CT scan. Sarcopenia has been shown to aid in predicting post-EmLap outcomes in various studies [21, 37–40]. The measurement after training can be simple, cost-effective, and reliable without need for additional advanced technology. Though this study found sarcopenia to not predict short and long-term outcomes, this could be due to the low number of patients included for the analysis, and the small mortality rates seen in the study cohort which may have limited the statistical power. The practicalities of assessing sarcopenia routinely in acute settings is not clearly demonstrated, as most of the sarcopenia measurements are done for research or study purposes.

According to Eight NELA report, approximately 55% of all EmLap operations in UK are patients over 65 years [34]. Although older adults were more likely to be frail and sarcopenic, this study found frailty (4.6%) and sarcopenia (22.2%) in younger adult (age under 65) population undergoing EmLap. This supports the results reported by Smart et al. (2017) which found frailty in up to 16% of younger adults admitted to emergency surgical units [3]. An analysis by Hanlon et al. (2018) using UK biobank participants reported that frailty is prevalent among middle-aged population (aged 37–65 years), with 3% of this group being frailty and 38% meeting criteria for pre-frailty [41]. Despite the impact of frailty in younger adult population and its potential importance, studies assessing frailty have often excluded patients aged under 65 years [42]. In clinical practice, methods to assess frailty have almost exclusively focused on older adult population [4]. 

Although there exists an overlap between frailty, sarcopenia, and poor physical status in this population, it is evident that one can have exclusively one factor, a combination of two factors or all three factors at once. The overlap may be contributed to underlying mechanisms such as shared risk factors, for example multimorbidity, socioeconomic status, and lifestyle factors [41]. While this study has explored the short and long-term mortality rates, further research with bigger sample size is required to investigate the impact of presence of one, two, or all three factors affect functional outcomes such as mobility, independence in daily activities and overall quality of life.

It is important to acknowledge several limitations of this study. Firstly, there were a small number of patients with 30-day mortality (3.7%), which is lower than the NELA 2023 where the report found overall 30-day mortality for patients undergoing EmLap laparotomy at 9.2%.^(34)^ This insufficient number may affect mortality prediction and limit the statistical power to calculate significance in the results. Secondly, due to small sample size, patients who are frail and pre-frail were incorporated in one group for the purposes of this paper. This may confound the statistical significance of frailty in EmLap settings. Lastly, this is a double-centre study, and future work should aim to involve more centres to allow better diversity in patient population in terms of type of EmLap, ethnicity, and socioeconomic backgrounds. This sample has been taken from a 96% Caucasian base Scottish population, which may prove difficult to apply this study’s results to other population groups as frailty and sarcopenia varies by country and ethnicity.

## Conclusion

Frailty, sarcopenia, and poor physical status are prevalent in patients undergoing emergency laparotomy. Frailty and poor physical status are associated with higher 30-day, 90-day and 1-year mortality risks compared to non-frail patients and those with normal physical status. Conversely, sarcopenic patients did not show a significant association with mortality risk over these time frames. While overlap exists between frailty, sarcopenia, and poor physical status, more understanding is required to understand the complex interplay.

## Electronic supplementary material

Below is the link to the electronic supplementary material.

Supplementary Material 1

Supplementary Material 2

## Data Availability

No datasets were generated or analysed during the current study.
