# Supplementary material for: The influence between frailty, sarcopenia and physical status on mortality in patients undergoing emergency laparotomy
Source: World J Emerg Surg. 2025 Apr 30;20:38. doi: 10.1186/s13017-025-00588-5 (PMC12042329; doi:10.1186/s13017-025-00588-5)
Supplement: Supplementary file 1 — Supplementary Material 1 [file 13017_2025_588_MOESM1_ESM.docx]

Additional file – Figure 1: Scatterplots assessing (a) inter- and (b) intra-observer variability. R^2^ line of fit should be close to 1.

**
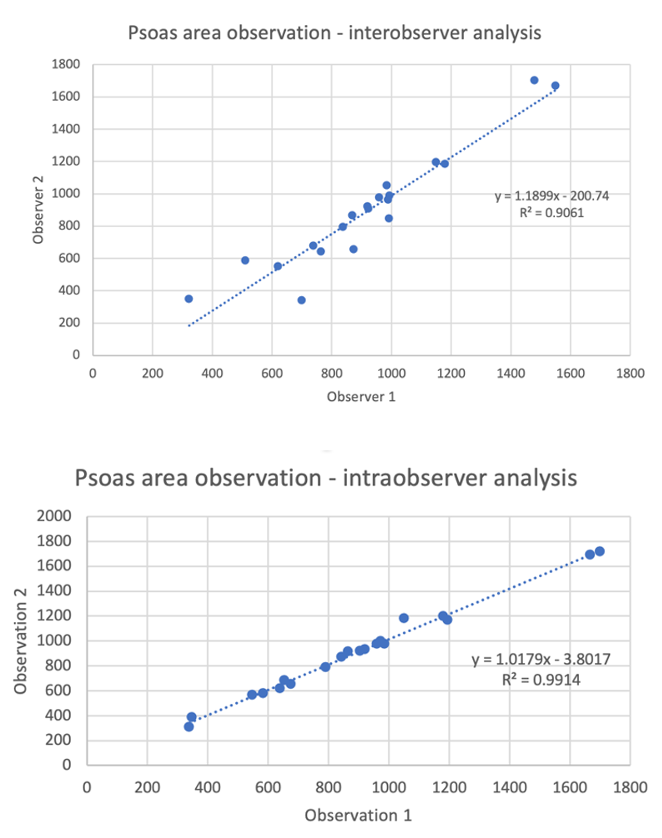
**

(b))

(a)
