# Supplementary material for: The influence between frailty, sarcopenia and physical status on mortality in patients undergoing emergency laparotomy
Source: World J Emerg Surg. 2025 Apr 30;20:38. doi: 10.1186/s13017-025-00588-5 (PMC12042329; doi:10.1186/s13017-025-00588-5)
Supplement: Supplementary file 2 — Supplementary Material 2 [file 13017_2025_588_MOESM2_ESM.docx]

Additional File– Table 1: Types of emergency laparotomy underwent by the total cohort.

| **Procedure** | **n (%)** |
| --- | --- |
| Colectomy: Right (Including ileocaecal resection)  Colectomy: Left  Colectomy: Subtotal or panproctocolectomy  Hartmann’s Procedure  Small bowel resection  Adhesiolysis  Stoma formation  Other | 42 (19.5%)  9 (4.2%)  10 (4.7%)  34 (15.8%)  41 (19.1%)  29 (13.5%)  22 (10.2%)  28 (13.0%) |
